# Assessment of the Effects of Edible Microalgae in a Canine Gut Model

**DOI:** 10.3390/ani12162100

**Published:** 2022-08-17

**Authors:** Costanza Delsante, Carlo Pinna, Federica Sportelli, Thomas Dalmonte, Claudio Stefanelli, Carla G. Vecchiato, Giacomo Biagi

**Affiliations:** 1 Department of Veterinary Medical Sciences, University of Bologna, Via Tolara di Sopra 50, Ozzano dell’Emilia, 40064 Bologna, Italy; 2 Department for Life Quality Studies, University of Bologna, Corso d’Augusto 237, 47921 Rimini, Italy

**Keywords:** dog intestinal microbiota, microalgae, canine nutrition, in vitro fermentation

## Abstract

**Simple Summary:**

The gut microbiota plays a crucial role in maintaining host health and is considered a potential target of novel therapeutics. Microalgae represent an interesting source of bioactive compounds for nutritional supplementation in humans and animals. However, there is a lack of information on the effects of microalgae on canine gut microbiota. The present study investigated for the first time the effects of four microalgae (Arthrospira platensis, Haematococcus pluvialis, Phaeodactylum tricornutum, Chlorella vulgaris) on some fecal bacterial populations and metabolites, such as SCFA, BCFA, ammonia and biogenic amines, in an in vitro canine gut model. Following the in vitro fermentation, chemical and microbiological analysis displayed significant differences between the control and microalgae groups, particularly the Phaeodactylum tricornutum group. Nonetheless, further investigations are needed to better understand the potential inclusion of these notable natural resources in pet nutrition.

**Abstract:**

Microalgae are a source of bioactive compounds having recently been studied for their possible application as health-promoting ingredients. The aim of the study was to evaluate in an in vitro canine gut model the effects of four microalgae, Arthrospira platensis (AP), Haematococcus pluvialis (HP), Phaeodactylum tricornutum (PT) and Chlorella vulgaris (CV), on some fecal microbial populations and metabolites. The four microalgae were subjected to an in vitro digestion procedure, and subsequently, the digested biomass underwent colonic in vitro fermentation. After 6 h of incubation, PT increased propionate (+36%) and butyrate (+24%), and decreased total BCFA (−47%), isobutyrate (−52%) and isovalerate (−43%) and C. hiranonis (−0.46 log10 copies/75 ng DNA). After 24 h, PT increased propionate (+21%) and isovalerate (+10%), and decreased the abundance of Turicibacter spp. (7.18 vs. 6.69 and 6.56 log10 copies/75 ng DNA for CTRL vs. PT, respectively); moreover, after 24 h, CV decreased C. coccoides (−1.12 log10 copies/75 ng DNA) and Enterococcus spp. (−0.37 log10 copies/75 ng DNA). In conclusion, the microbial saccharolytic activities and the shift in fecal bacterial composition were less pronounced than expected, based on current literature. This study should be considered as a preliminary assessment, and future investigations are required to better understand the role of microalgae in canine nutrition.

## 1. Introduction

The gastrointestinal tract of mammals harbors one of the largest and most complex ecosystems known, including numerous bacterial species, along with fungi, protozoa and viruses. Over the past decades, there has been a growing understanding that the gut microbiota profoundly contributes to improve host health [1]. For example, intestinal bacteria either generate or turn dietary molecules into bacteria-derived metabolites, and the gut microbiome is considered an important metabolic organ. A well-balanced gut microbiome positively affects host health by modulating immune system, exerting protective action against intestinal pathogens, and providing some nutrients [2]. Among the bacterial metabolites, straight short-chain fatty acids (SCFA) acetate, propionate and butyrate and branched-chain fatty acids (BCFA) isobutyrate and isovalerate [3] exercise very different but important activities in the host body [4]. In particular, SCFA from microbial metabolism enhance the function of the intestinal barrier, regulating the luminal pH and the mucus secretion, modulating the mucosal immune function and providing a source of energy for colonocytes [5].

In this vein, modulation of gut microbiota should be taken into consideration as a possible novel therapeutic [6].

The diet plays an important role in shaping the composition of intestinal microbiota and its relation with the host [6]. In the last decades, scientific research has widely investigated different nutritional strategies with the purpose of positively affecting the microbial ecosystem of canine gastrointestinal tract [7]. Among the several dietary components investigated in this context, edible microalgae, also called microphytes, represent an interesting source of bioactive compounds including protein, polysaccharides, polyunsaturated fatty acids, pigments, minerals, phenolic compounds, vitamins, volatile compounds and sterols, hence offering several possible health benefits [8]. Microalgae are ancient living organisms belonging to a taxonomically heterogeneous group, which includes organisms from a series of diverse classes and phyla [9]; some cyanobacteria belong to microalgae as well [8]. Among the high number of microalgae species in nature, Arthrospira and Chlorella are the algal strains more presented on the market worldwide [10]. Arthrospira has a global annual production of 12,000 tons, followed by Chlorella with about 5000 tons per year [11], and both genera are commercially available for human consumption, in accordance with the Food and Drug Administration [12], being found on the market as functional foods [13]. Furthermore, dried algae, algal oil, algae meal and extract are listed in the European catalogue of feed materials [14]. Arthrospira and Chlorella are rich in unsaturated fatty acids, proteins, various minerals, group B vitamins and pigments with relevant antioxidant characteristics, and thus, they may be used in foods [15,16]. Apart from the interest for the whole dried algae, currently, specific high-value constituents from microphytes are also being investigated. In this domain, Haematococcus pluvialis and Phaeodactylum tricornutum are two microphyte species with relevant bioactive compounds [13]. The first is cultured to obtain astaxanthin (a carotenoid with antioxidant properties) [17], while the second is a marine diatom contemplated as a significant potential source of eicosapentanoic acid (EPA) and carotenoids [18].

For their properties, microphytes have been suggested to be a promising sustainable alternative to traditional animal feed resources and a possible health-promoting ingredient both in human diet [15,17] and animal feeds [19], particularly in livestock and poultry feed industry [20,21], and in the aquaculture field [22,23]. However, microalgae supplementation requires a clear understanding of their effects on the intestinal microbiota and bacterial metabolome of the host.

In recent years, studies have identified many positive benefits of microalgae, including immunomodulatory [24,25], antioxidant [26], anti-inflammatory [27,28] and anti-bacterial effects [29]. In addition, some microalgae are also known to have prebiotic properties [30], thus modulating the gastrointestinal microbiota. Recently, De Medeiros et al. [31] investigated the prebiotic properties of some digested microalgae biomass, including Chlorella vulgaris and Arthrospira platensis, on human colonic microbiota during in vitro fermentation. Microalgae biomass showed a selective stimulation of beneficial microorganisms and inhibition of undesired bacteria on colonic microbiota. A previous study conducted by Jin et al. [32] reported that supplementation with Chlorella vulgaris and other microalgae increased propionate-producing bacteria in an in vitro human gut fermentation model. According to the previously cited studies, in order to evaluate the effects of microalgae on intestinal microbiota, it is appropriate to use an in vitro digestion procedure and subsequently undergo the digested biomass to colonic in vitro fermentation, with the aim to examine the potential microbiota modulation activities of microalgae [31,32].

It must be emphasized that very few studies have investigated the use of microphytes in dogs and they were mainly focused on anti-inflammatory and immunomodulating activities of whole microalgae or specific components. Cortese et al. [33] investigated the immune-modulating effect of a specific diet in canine Leishmaniosis. Dogs were given an anti-Leishmania drug therapy combined with a standard diet or with the specific immune-modulating diet containing some nutraceutical substances including Astaxanthin from Haematococcus pluvialis. The immune-modulating diet appeared to control the immune response in canine Leishmaniosis during the standard pharmacological therapy. In fact, the immune-modulating diet decreased T-helper 1 cells inflammatory response and increased regulatory T cells in diseased dogs. It must be underlined that the dosage of astaxanthin and other individual nutraceutical substances was not reported in the study. For that reason, it is not possible to discriminate their effect separately. More recently, Satyaraj et al. [25] evaluated the immunomodulatory effect of dietary supplementation with 0.2% spray-dried Arthrospira platensis in dogs. Dogs fed diets supplemented with Arthrospira showed enhanced immune status and higher levels of fecal IgA as compared to the control group. It has been shown that fecal concentration of IgA decreases in dogs with chronic enteropathies, and thus, fecal IgA level in dogs may be considered as an indicator of intestinal immune health [34,35]. Furthermore, this investigation demonstrated that supplementing diets with Spirulina also resulted in significantly increased gut microbiota stability [25].

However, to our knowledge, there are no studies investigating the effects of microphytes on canine gut microbiota and concentrations of bacterial metabolites such as SCFA, BCFA, ammonia and biogenic amines, which are known to be of fundamental relevance in host–microbial interactions [36].

The aim of the present study was to evaluate in an in vitro canine gut model the effects of four microalgae (Micoperi Blue Growth, Ravenna, Italy) Arthrospira platensis (AP), Haematococcus pluvialis (HP), Phaeodactylum tricornutum (PT) and Chlorella vulgaris (CV) on some fecal microbial populations and metabolites. We selected these four species because they currently represent the microalgae of greatest interest in the food and feed sector [8,13]. We hypothesized that composition and metabolism of canine fecal microbiota would be influenced by microalgae supplementation; particularly, we expected an increase concentration of the three major SCFA (acetate, propionate and butyrate), in accordance with previously in vitro cited studies [31,32], and higher concentration of microbial species known to be SCFA producers (i.e., Ruminococcaceae, Clostridium cluster XIV, Turicibacter spp., C. leptum, C. coccoides) [37]; in addition, we assumed that microbial species thought to have undesired influences on canine gut health would decrease. Lastly, we hypothesized a decrease of metabolites deriving from bacterial proteolysis (ammonia, BCFA, biogenic amines) due to the enhanced saccharolytic activities of bacterial populations. To the authors’ knowledge, this is the first study considering the effects of microalgae on proteolytic microbial activities.

## 2. Materials and Methods

### 2.1. Experimental Set-Up

Preliminarily, the microalgae were subjected to in vitro digestion, in order to simulate the digestion processes that take place in the stomach and small intestine of dogs, according to the method proposed by Biagi et al. [38]. Briefly, the samples undergo two incubation phases, a first lasting 2 h and taking place in the presence of pepsin, gastric lipase and HCl (gastric phase) and a second 4 h one with phosphate-bicarbonate buffer, pancreatin and bile salts (intestinal phase). Proximate analysis of the four microalgae, their undigested fraction and the digestibility coefficients are reported in Table 1. In this experiment, the undigested fraction of each microalga was tested as a fermentation substrate.

Five healthy adult dogs (mixed breed; average body weight of 21 kg; age 3.6 years), privately owned, were fed the same commercial dry diet for adult dogs (Agras Delic Spa, Genova, Italy) for 28 d. The diet contained the following ingredients: corn, barley, dehydrated venison, potato protein, purified pork fat, dried beet pulp, sunflower oil, brewer’s yeast, dried chicory pulp, FOS, cod liver oil, dicalcium phosphate, potassium chloride, sodium chloride, herbs (dog rose, bearberry, blackcurrant, taraxacum and thistle) and Yucca schidigera. The proximate composition of the diet (on dry matter basis) was the following: crude protein (CP) 23.6%, ether extract (EE) 12.5%, crude ash (ash) 5.71%, starch 38.9% and crude fiber (CF) 2.08%.

The same dry food that was fed to the dogs used as fecal donors was subjected to in vitro digestion using the same procedure used for the microalgae. After in vitro digestion, the recovery rate of the diet was 18.5% (on dry matter basis) and the chemical composition of the undigested residue (on dry matter basis) was the following: CP 17.3%, EE 2.43%, starch 3.87%, ash 14.6% and CF 9.94%.

After the 28-day feeding period, a sample of fresh feces was collected from each dog immediately after defecation and delivered to the laboratory. Within one hour from the excretion, the feces were pooled and suspended at 10 g/L in prereduced Wilkins Chalgren anaerobe broth. The fecal suspension was used to inoculate (100 mL/L) a previously warmed (39 °C) and prereduced medium prepared according to Sunvold et al. [39]. Five vessels (30 mL) were arranged per treatment.

Five treatments were arranged: (1) control diet with no addition of experimental substrates and control diet with (2) Arthrospira platensis, (3) Haematococcus pluvialis, (4) Phaeodactylum tricornutum, or (5) Chlorella vulgaris. All vessels contained the undigested residue of the commercial dry food for dogs at 10 g/L. The amount of microalgae that was added to the inocula is reported in Table 2. Amounts were calculated based on the different in vitro digestibility coefficients of microalgae (Table 1). The dose that was used should reflect the amount of microalgae that reach the hindgut when they are included in a commercial extruded food for dogs (with a digestibility of approximately 90%) at a concentration of 40 g/kg.

The pH of fecal cultures was adjusted to 6.7; bottles were sealed and incubated for 24 h at 39 °C under an 85% N_2_, 10% CO_2_ and 5% H_2_ atmosphere. Samples of inocula were collected from each vessel at 6 and 24 h for the determination of pH, ammonia, biogenic amines, SCFA and for microbial analysis.

### 2.2. Chemical Analyses

The commercial dry food, the algae and their undigested residue were analyzed according to the AOAC International standard methods (method 950.46 for water, method 954.01 for CP, method 920.39 for EE, method 920.40 for starch, method 942.05 for ash and method 962.09 for CF) [40]. Ammonia was determined by an enzymatic colorimetric procedure (Urea/BUN—Color; BioSystems S.A., Barcelona, Spain) [41]. The SCFA and BCFA were separated using 10% SP-1000 + 1% H_3_PO_4_, 100/120 mesh Chromosorb W AW in a 1.8-m by 2-mm (internal diameter) glass column, with nitrogen as the carrier [42]. The chromatograph was a Fisons HRGC MEGA 2 series 8560 with a flame ionization detector. The temperatures of the injector and detector were 200 °C, and the oven temperature was 155 °C. 2-ethylbutyric acid was used as the internal standard. For the determination of biogenic amines, samples were diluted 1:5 *w*/*v* with perchloric acid (0.3 M); biogenic amines were later separated by HPLC and quantified through fluorimetry [43].

### 2.3. Microbial Analysis

At each sampling time, a 1 mL portion of fermentation fluid was collected from each vessel and centrifuged at 4 °C for 5 min, at 18,000× *g*. The supernatant was removed and immediately frozen at −80 °C for further analysis. Bacterial genomic DNA was extracted from remaining pellet using the Stool DNA isolation kit (Norgen Biotek Co., Thorold, ON, Canada). Isolated DNA concentration (ng/µL) and purity were measured using a DeNovix DS-11 spectrophotometer (DeNovix Inc., Wilmington, DE, USA). Template DNA was diluted to 50 ng/µL and stored at −20 °C until further analysis. Turicibacter, Ruminococcaceae, Blautia, *Escherichia coli*, *Bifidobacterium* spp., *Lactobacillus* spp., *Enterococcus* spp., *Clostridium* cluster XIV, *Clostridium coccoides*, *Clostridium leptum* and *Clostridium hiranonis* were quantified via quantitative polymerase chain reaction (qPCR) using specific primers. Detailed information of primers is presented in Table 3.

Reaction mixtures were prepared in 15-μL volumes containing 7.5 µL 2XSensiFAST No-ROX PCR MasterMix (Meridian Bioscience Inc., Cincinnati, OH, USA), 4.8 µL of nuclease-free water, 0.6 µL of each 10 pmol primer and 1.5 µL of sample DNA. The assay consisted of a 2-min denaturation at 95 °C, followed by 40 cycles of 95 °C for 5 s, primer annealing at 56–66 °C for 10 s and 72 °C for 8 s. The cycle was repeated 40 times. A negative control (without the DNA template) was also run for each primer pair. The qPCR assay was performed using a CFX96 Touch thermal cycler (Bio-Rad, Hercules, CA, USA). Amplification was performed in duplicate for each bacterial group within each sample, while standard curves were run in triplicate. Standard curves were constructed from eight tenfold dilutions for each target. Cycle threshold values were plotted against standard curves for the quantification of the target bacterial DNA from fecal inoculum. Melting curves were checked after amplification to ensure the single product amplification of a consistent melting temperature.

### 2.4. Statistical Analyses

Kruskal–Wallis one-way ANOVA with Dunn’s multiple comparisons were performed for data with unequal variances, while normally distributed data were compared using a one-way ANOVA with Dunnett’s multiple comparison test. Differences between groups were considered significant for *p* < 0.05. Each vial represented a single experimental unit. Significance and tendency for statistical tests were set at *p* < 0.05 and 0.05 < *p* < 0.1, respectively. Statistical analyses were performed using Statistica 10.0 software (Stat Soft Italia, Padua, Italy).

## 3. Results

The chemical parameters evaluated on samples of fermentation fluid collected after 6 and 24 h of incubation are shown in Table 4 and Table 5, respectively. After 6 h of incubation, pH was decreased by HP, PT and CV compared to CTRL (6.58, 6.63 and 6.56 vs. 6.71, respectively; *p* < 0.05). Conversely, after 24 h of incubation, pH values were not affected by treatments (*p* > 0.05). Moreover, the concentration of ammonia did not change after 6 and 24 h of incubation. Total concentrations of SCFA were not influenced by treatments after 6 and 24 h. On the contrary, total BCFA were decreased in flasks containing PT and CV (−46% for both; *p* < 0.05) at 6 h; however, this effect was no longer present after 24 h. At 6 h, flasks with PT contained higher concentration of propionate (+36%; *p* < 0.05) and butyrate (+24%; *p* < 0.05). Additionally, propionate proportions were higher in all the flasks treated with microalgae, both at 6h (+2.6% for AP; +2.8% for HP; +6.2% for PT; +3.5% for CV; *p* < 0.05) and 24 h (+1.1% for AP; +1.4% for HP; +3.9% for PT; +1.9% for CV; *p* < 0.05). After 6 h of incubation, isobutyrate was reduced by PT (−52%; *p* < 0.05) and isovalerate was decreased by all treatments except HP (−43% for AP, PT, CV; *p* < 0.05). At 24 h, propionate was still higher in vessels containing PT (+21%; *p* < 0.05), while BCFA were not affected by microalgae with the exception of isovalerate concentration that was higher in PT (+10%; *p* < 0.05). In addition, no significant effects were observed in regard to biogenic amines both at 6 and 24 h, as reported in Table 6.

**Figure 1:**
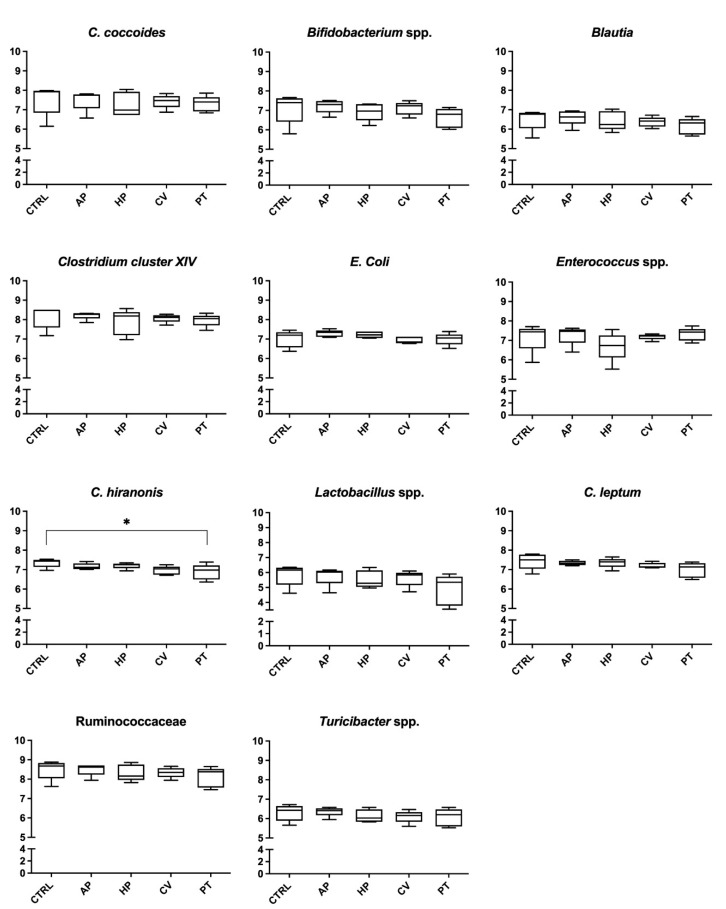
Microbial analysis (log_10_ copies DNA/75 ng DNA) after 6 h of an in vitro incubation of canine fecal inoculum with a control diet supplemented with microalgae. Values are the means of five bottles per treatment. CTRL, control; AP, *Arthrospira platensis*; HP, *Haematococcus pluvialis*; CV, *Chlorella vulgaris*; PT, *Phaeodactylum tricornutum*. * *p* < 0.05.

**Figure 2:**
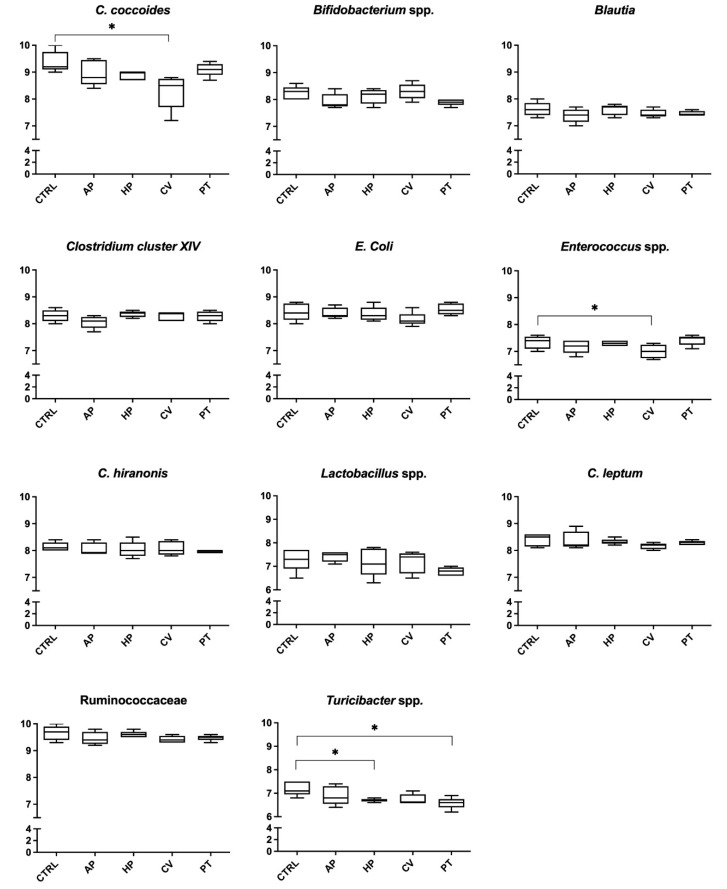
Microbial analysis (log_10_ copies DNA/75 ng DNA) after 24 h of an in vitro incubation of canine fecal inoculum with a control diet supplemented with microalgae. Values are the means of five bottles per treatment. CTRL, control; AP, *Arthrospira platensis*; HP, *Haematococcus pluvialis*; CV, *Chlorella vulgaris*; PT, *Phaeodactylum tricornutum*. * *p* < 0.05.

The data relating to the composition of the fecal microbiota evaluated at 6 and 24 h of incubation are presented in Figure 1 and Figure 2, respectively. After 6 h, treatments containing PT decreased the abundance of *C. hiranonis* (6.88 vs. 7.34 log_10_ copies/75 ng DNA; *p* < 0.05). Microphyte treatments decreased the DNA concentration of some bacterial populations after 24 h. In particular, *Turicibacter* spp. was reduced by HP and PT (6.69 and 6.56 vs. 7.18 log_10_ copies/75 ng DNA, respectively; *p* < 0.05). Finally, *C. coccoides* (8.26 vs. 9.38 log_10_ copies/75 ng DNA *p* < 0.05) and *Enterococcus* spp. (6.99 vs. 7.36 log_10_ copies/75 ng DNA *p* < 0.05) were less abundant in flasks containing CV.

## 4. Discussion

The purpose of this investigation was to assess the in vitro effects of four microalgae on some canine fecal microbial populations and metabolites.

In this study, supplementation with microalgae partially affected the gut ecology. In particular, pH was decreased after 6 h in three out of four microalgae groups (HP, CV, PT). Changes in pH can affect microbial communities, thus impacting the concentration and profile of fermentation products [53]. Particularly, the reduction of intestinal pH could be a desirable effect, as the acidification of the environment has a broad-spectrum inhibitory activity against Gram-positive and Gram-negative bacteria. At the same time, it is known how the colonic pH is influenced by fermentation processes of bacterial populations, in particular in the proximal colon, where the pH is lower due to the production of SCFA that mainly derive from the fermentation of carbohydrates [54,55]. However, in the present investigation, total concentration of SCFA was not affected by the treatments.

After 6 h of incubation, the concentration of propionate and butyrate was increased by PT. A previous study conducted in an in vitro human gut model demonstrated that supplementation with digested microphytes (2.5 g/dL) could affect both intestinal microbiota composition and metabolites [32]. In that study, Jin et al. [32] investigated the effects of three edible microalgae (*Chlorella vulgaris*, *Chlorella protothecoides* and *Schizochytrium* sp.) on human gut microbiota, showing that microalgae supplementation increased the proportion of propionate in the colonic culture together with the relative abundance of some bacterial populations involved in propionate metabolism (genera *Bacteroides* spp. and *Dialister* spp.). Moreover, total SCFA were significantly increased by *C. vulgaris*. Similar effects, although to a minor extent, were observed in the present study regarding the higher concentration and proportions of propionate in PT group, both after 6 and 24 h. Intestinal SCFA are linked with some health-promoting effects, such as anti-inflammatory, immune-regulatory and anticarcinogenic functions [56]. Specifically, propionate is metabolized in the liver and plays a role in reducing the concentration of blood sugar and serum cholesterol, while butyrate is an important source of energy for colonocytes [57]. In addition, butyrate is known to be responsible for functions associated with the gut microbiota and physiology, maintaining cell growth and differentiation. It also plays a role in the prevention of colonic cancer and in the reduction of inflammation [58].

Nevertheless, in the present trial higher concentration of some SCFA did not reflect a change in microbial populations known as SCFA producers. Moreover, after 24 h of fermentation, lower abundance of *Enterococcus* spp. and *C. coccoides* (in CV vessels) and *Turicibacter* spp. (in PT and HP vessels) were detected in three of the groups to which microalgae were added. These last outcomes are in contrast with the results recently obtained by Wan et al. [59], who studied the effect of a bioactive polysaccharide from microalga *Chlorella pyrenoidosa* (CPP) at a dosage of 150 and 300 mg/kg, on gut microbiota of mice fed a high-fat diet. The authors pointed out that the growth of some bacterial genera, including *Turicibacter*, and the concentrations of acetate, propionate and butyrate were drastically increased in both CPP treatments. *Turicibacter* spp., belonging to the Firmicutes phylum, is considered an important producer of SCFA [60], suggesting an important role of *Turicibacter* spp. in promoting gut health.

In relation to the decrease of *C*. *coccoides* in the CV group observed in the present study after 24 h, existing outcomes regarding the role of this bacterial species in host physiology appear to be controversial. *C. coccoides* is a member of the Firmicutes phylum, one of the most preponderant bacterial populations in the human intestine, and many species in this group, such as *Eubacterium* spp., *Roseburia* spp., *Subdoligranulum variabile* and *Faecalibacterium prausnitzii*, directly produce butyrate from dietary polysaccharides and other substrates [61]; moreover, changes in relative abundance of the Firmicutes are also associated with an increased ability to harvest energy from diet [62]. In the literature, the decrease in abundance of the *E. rectale-C. coccoides* group (Clostridium cluster XIVa and XIVb) has also been correlated with the presence of phenolic compounds with inhibitory effects on the growth of potentially adverse bacteria [63]. Phenolic compounds are a wide group of secondary metabolites encompassing phenolic acids, flavonoids, isoflavonoids, stilbenes, lignans and phenolic polymers. These molecules show a broad spectrum of biological activities comprising antioxidant activities as well as anti-inflammatory, anti-cancer, anti-allergic, anti-diabetes, anti-aging and antimicrobial properties [64]. High levels of phenolic compounds with antioxidant activity are present in microalgae, and like other active biological compounds, both the composition and content of polyphenol from microalgae are species-dependent [11]. According to Goiris et al. [65], *Phaeodactylum tricornutum* is the microalga that contains the greatest number of polyphenols [375 mg Gallic Acid Equivalent (GAE)/100 g DW, against the 54 mg (GAE)/100 g DW from *Haematococcus pluvialis*], while *Chlorella vulgaris* exhibits 221 mg GAE/100 g DW. Microalgae polyphenols content could be linked with the lower abundances of *C. coccoides* in CV group after 24 h of fermentation observed in the present study. However, in the present investigation, the phenolic compounds content of the four microalgae was not evaluated, and for that reason, it is not possible to discuss its role in modulating microbial population.

The decrease in *E. rectale-C. coccoides* group relative abundances were also observed by de Medeiros et al. [31] during their in vitro study with a human colonic model. The 48 h fermentation was performed in batches composed of 40% of a fermentation medium, 40% of pooled human fecal inoculum and 20% of a digested microalgae biomass, including *Chlorella vulgaris* and *Arthrospira Platensis*. In the same experiment, digested microalgae biomass increased the *Lactobacillus-Enterococcus* and *Bifidobacterium* spp. relative abundance. On the contrary, in the present investigation, microbial analysis showed that, at 24 h, CV treatment decreased enterococci, considered as members of the healthy intestinal microbiota and potentially probiotic. Enterococci have different features such as auto- and co-aggregation ability, tolerance to low pH and bile salts, adherence to hydrocarbons, sensibility to some antibiotics, as well as exo-polysaccharide synthesis [66].

After 6 h of incubation, PT resulted in decreased abundance of *C. hiranonis*. *C. hiranonis* is a bacterial species of interest, as it shows bile acid 7 alpha-dehydroxylating activity, and a decrease in *C. hiranonis* may suggest bile acid dysmetabolism [50]. These findings are apparently in contrast with previously mentioned studies [31,32], in which microphytes seemed to enhance intestinal health by stimulating the growth of beneficial bacterial population, such as SCFA-producing bacteria. Certainly, it must be underlined that, in the present study, only a few of the main populations of canine microbiota have been evaluated by quantitative PCR. This fact represents a limitation, as we cannot exclude that any changes regarding other bacteria could not have been detected.

One of the main reasons for considering microalgae as an interesting source of food is their high protein content (e.g., 55–70% for *S. platensis* and 42–55% for *C. vulgaris* on a dry matter basis [67]). In this study, microalgae were preliminarily subjected to in vitro digestion and the undigested fraction was used as the fermentation substrate. Proteins were highly represented in the undigested fraction of AP (55.7%), CV (18.0%) and PT (18.6%). Interestingly, the presence of microalgae, despite the increased presence of protein, decreased BCFA after 6 h and did not result in higher concentrations of ammonia and biogenic amines, metabolites deriving from bacterial proteolysis [36,68]. In particular, PT seemed to have the greatest effect on BCFA by decreasing both the concentration of isobutyrate and isovalerate. BCFA are synthetized in the colon from bacterial metabolism of branched-chain amino acids valine, leucine and isoleucine [69]. According to Safi et al. [70], HP resulted higher in branched-chain amino acids when compared to AP. Therefore, despite the higher amount of crude protein in AP vessels, we speculate that differences in isovalerate concentrations could be associated to the different amino acids’ composition of the substrates. The biological significance of BCFA and biogenic amines is still poorly understood. It has been hypothesized that BCFA may have a role in the regulation of ionic exchanges in colonic mucosa [71] and that isobutyrate may act as a potential source of energy for colonocytes after exhaustion of butyrate [72]. Similarly, biogenic amines seem to have a beneficial influence on the intestinal mucosa [73]; however, on the other hand, they could act as precursors in the formation of nitrosamines, known as carcinogens in humans [74]. The decrease of BCFA that we observed could indicate a reduction of proteolytic activities operated by some bacterial populations. However, other parameters, including concentration of ammonia and biogenic amines, did not reflect this trend. In this regard, the effects of microalgae supplementation on metabolites deriving from bacterial proteolysis are still poorly investigated, and hence, it could represent an interesting aspect to be explored.

## 5. Conclusions

During the present in vitro study, microalgae partially affected canine fecal microbiota. Among the four microphytes, PT showed the greatest effect on microbial metabolites after 6 h of incubation by increasing propionate, butyrate and decreasing BCFA. These outcomes suggest that microalgae, especially PT, could have a potential modulatory effect on the metabolic activities of canine intestinal microorganisms. However, PT led to a reduction of *C. hiranonis* at 6 h, while after 24 h, HP, CV and PT resulted in a decrease of some beneficial bacterial populations belonging to Firmicutes, known to be butyrate-producing bacteria.

The present study has led to unexpected results, as we would have presumed a more pronounced microbial saccharolytic activities and a greater shift in fecal bacterial composition. Therefore, recommendation of microalgae as food supplements required more research on the effects of their dietary inclusion. Thus, our work should be considered as a preliminary study for future investigations.

## Figures and Tables

**Table 1:** Proximate analysis of four microalgae and their undigested fraction, and digestibility coefficients of microalgae subjected to in vitro digestion. Values are reported as % on dry matter basis.

Item	Crude Protein	Crude Ash	Crude Fibre	Total Digestibility
Microalgae				
*Arthrospira platensis*	70.9	5.03	0.70	86.2
*Haematococcus pluvialis*	10.4	4.03	15.67	7.87
*Phaeodactylum tricornutum*	39.6	22.4	0.40	67.5
*Chlorella vulgaris*	31.1	9.97	11.8	55.3
Microalgae, undigested fraction				
*Arthrospira platensis*	55.7	4.04	13.7	
*Haematococcus pluvialis*	10.2	1.53	9.84	
*Phaeodactylum tricornutum*	18.6	27.0	0.56	
*Chlorella vulgaris*	18.0	10.7	16.8	

**Table 2:** Amount of undigested fraction of the commercial dry food and microalgae that were added to each bottle. Each bottle contained 21 mL of fecal culture.

Treatment	Commercial Dry Food, Undigested Fraction (mg)	Algae, Undigested Fraction (mg)
Control (CTRL)	210	-
*Arthrospira platensis* (AP)	210	11.6
*Haematococcus pluvialis* (HP)	210	77.4
*Phaeodactylum tricornutum* (PT)	210	37.5
*Chlorella vulgaris* (CV)	210	27.3

**Table 3:** Primers used in the qPCR assay.

Target	Primer	Sequence (5′→3′)	Annealing Temperature (°C)	Reference
*Blautia* spp.	Blautia_F	TCTGATGTGAAAGGCTGGGGCTTA	62.0	[44]
	Blautia_R	GGCTTAGCCACCCGACACCTA		
*Turicibacter* spp.	Turicibacter_F	CAGACGGGGACAACGATTGGA	59.3	[44]
	Turicibacter_R	TACGCATCGTCGCCTTGGTA		
Ruminococcaceae	Ruminococcaceae_F	ACTGAGAGGTTGAACGGCCA	64.2	[45]
	Ruminococcaceae_R	CCTTTACACCCAGTAAWTCCGGA		
*Bifidobacterium* spp.	Bif_F	TCGCGTCYGGTGTGAAAG	62.0	[46]
	Bif_R	CCACATCCAGCRTCCAC		
*Lactobacillus* spp.	Lac_F	AGCAGTAGGGAATCTTCCA	64.2	[47]
	Lac_R	CACCGCTACACATGGAG		
*Clostridium leptum*	sg-Clept-F	GCACAAGCAGTGGAGT	59.3	[48]
	sg-Clept-R	CTTCCTCCGTTTTGTCAA		
*Clostridium coccoides*	g-Ccoc-F	AAATGACGGTACCTGACTAA	64.2	[49]
	g-Ccoc-R	CTTTGAGTTTCATTCTTGCGAA		
*Clostridium hiranonis*	C.hiranonis_F	AGTAAGCTCCTGATACTGTCT	65.4	[50]
	C.hiranonis_R	AGGGAAAGAGGAGATTAGTCC		
*Escherichia coli*	Coli_F	GTTAATACCTTTGCTCATTGA	62.0	[51]
	Coli_R	ACCAGGGTATCTAATCCTGTT		
*Enterococcus* spp.	Ent_F	CCCTTATTGTTAGTTGCCATCATT	59.3	[46]
	Ent_R	ACTCGTTGTACTTCCCATTGT		
*Clostridium* cluster XIV	CloXIV-F	GAWGAAGTATYTCGGTATGT	57.2	[52]
	CloXIV-R	CTACGCWCCCTTTACAC		

**Table 4:** pH values, ammonia and short-chain fatty acids concentrations after 6 h of an in vitro incubation of canine fecal inoculum supplemented with microalgae ^1^.

Item	CTRL	AP	HP	PT	CV	Pooled SEM	Anova *p*-Value
pH	6.71	6.63	6.58 *	6.63 *	6.56 *	0.03	0.005
Ammonia, mmol/L	30.2	32.2	31.4	29.6	31.9	1.62	0.586
Straight-chain SCFA, mmol/L							
Acetate	8.62	8.66	8.97	8.85	8.57	0.42	0.954
Propionate	4.54	4.92	5.13	6.19 *	5.14	0.23	0.001
Butyrate	2.55	2.58	2.62	3.16 *	2.69	0.12	0.013
Total SCFA	15.7	16.2	16.7	18.2	16.4	0.78	0.232
BCFA, mmol/L							
Isobutyrate	0.27	0.15	0.15	0.13 *	0.13	0.03	0.022
Isovalerate	0.46	0.26 *	0.30	0.26 *	0.26 *	0.03	0.009
Total BCFA	0.73	0.41	0.45	0.39 *	0.39 *	0.08	0.006
Individual SCFA proportions, %							
Acetate	51.5	52.2	52.1	47.6 *	51.0	0.44	<0.001
Propionate	27.1	29.7 *	29.9 *	33.3 *	30.6 *	0.23	<0.001
Butyrate	15.2	15.5	15.2	17.0 *	16.0 *	0.15	<0.001
Isobutyrate	1.54	0.88	0.90	0.71 *	0.80 *	0.13	0.001
Isovalerate	2.70	1.59	1.74	1.42 *	1.56 *	0.13	<0.001

^1^ Values are the means of five bottles per treatment. * Significantly different from CTRL, *p* < 0.05. CTRL, control; AP, *Arthrospira platensis*; HP, *Haematococcus pluvialis*; PT, *Phaeodactylum tricornutum*; CV, *Chlorella vulgaris*; SCFA, short-chain fatty acid; BCFA, branched-chain fatty acid.

**Table 5:** pH values, ammonia and short-chain fatty acids concentrations after 24 h of an in vitro incubation of canine fecal inoculum supplemented with microalgae ^1^.

Item	CTRL	AP	HP	PT	CV	Pooled SEM	Anova *p*-Value
pH	5.84	5.84	5.81	5.95	5.81	0.01	0.004
Ammonia, mmol/L	39.6	39.9	36.0	38.0	35.7	1.29	0.065
Straight-chain SCFA, mmol/L							
Acetate	16.7	16.9	16.6	16.5	16.5	0.48	0.960
Propionate	9.68	10.5	10.3	11.7 *	10.7	0.28	0.001
Butyrate	5.43	5.73	5.31	5.65	5.61	0.14	0.271
Total SCFA	31.8	33.1	32.2	33.8	32.8	0.89	0.536
BCFA, mmol/L							
Isobutyrate	0.60	0.64	0.60	0.64	0.62	0.02	0.289
Isovalerate	0.92	0.95	0.90	1.01 *	0.94	0.02	0.041
Total BCFA	1.52	1.59	1.50	1.65	1.56	0.04	0.086
Individual SCFA proportions, %							
Acetate	48.7	47.3 *	47.9 *	45.2 *	46.7 *	0.20	<0.001
Propionate	28.3	29.4 *	29.7 *	32.2 *	30.2 *	0.09	<0.001
Butyrate	15.9	16.0	15.4 *	15.6	15.9	0.10	0.001
Isobutyrate	1.76	1.80	1.74	1.76	1.75	0.01	0.041
Isovalerate	2.68	2.66	2.59	2.78	2.65	0.05	0.254

^1^ Values are the means of five bottles per treatment. * Significantly different from CTRL, *p* < 0.05. CTRL, control; AP, *Arthrospira platensis*; HP, *Haematococcus pluvialis*; PT, *Phaeodactylum tricornutum*; CV, *Chlorella vulgaris*; SCFA, short-chain fatty acid; BCFA, branched-chain fatty acid.

**Table 6:** Biogenic amines concentrations (nmol/mL) 6 h and 24 h of an in vitro incubation of canine fecal inoculum with a control diet supplemented with microalgae ^1^.

Item	CTRL	AP	HP	PT	CV	Pooled SEM	Anova *p*-Value
*6 h*							
Putrescine	177.4	186.6	175.6	169.2	179.0	4.87	0.241
Cadaverine	101.0	124.6	132.4	87.4	96.4	15.1	0.371
Spermidine	24.4	68.8	36.4	21.6	23.6	9.55	0.043
Spermine	3.80	3.70	5.02	0.98	1.28	6.85	0.041
*24 h*							
Putrescine	166.4	174.0	111.4	140.4	107.8	10.2	0.007
Cadaverine	129.8	154.4	72.4	97.4	113.8	25.6	0.223
Spermidine	22.0	21.8	18.2	22.6	24.6	3.00	0.669
Spermine	1.32	1.16	1.04	0.58	2.12	0.53	0.414

^1^ Values are the means of five bottles per treatment. CTRL, control; AP, *Arthrospira platensis*; HP, *Haematococcus pluvialis*; PT, *Phaeodactylum tricornutum*; CV, *Chlorella vulgaris*.

## Data Availability

All data are available in the manuscript.
